# A Novel Fungal Lipase With Methanol Tolerance and Preference for Macaw Palm Oil

**DOI:** 10.3389/fbioe.2020.00304

**Published:** 2020-05-06

**Authors:** Letícia L. Rade, Melque N. P. da Silva, Plínio S. Vieira, Natalia Milan, Claudia M. de Souza, Ricardo R. de Melo, Bruno C. Klein, Antonio Bonomi, Heizir F. de Castro, Mário T. Murakami, Leticia M. Zanphorlin

**Affiliations:** ^1^Brazilian Biorenewables National Laboratory, Brazilian Center for Research in Energy and Materials, Campinas, Brazil; ^2^Department of Chemical Engineering, Engineering School of Lorena, University of São Paulo, Lorena, Brazil

**Keywords:** biohydrocarbons, “drop-in” biofuels, mechanistic enzyme, *Acrocomia aculeate*, fungal lipase, macaw oil

## Abstract

Macaw palm is a highly oil-producing plant, which presents high contents of free fatty acids, being a promising feedstock for biofuel production. The current chemical routes are costly and complex, involving highly harsh industrial conditions. Enzymatic processing is a potential alternative; however, it is hampered by the scarce knowledge on biocatalysts adapted to this acidic feedstock. This work describes a novel lipase isolated from the thermophilic fungus *Rasamsonia emersonii* (*Re*Lip), which tolerates extreme conditions such as the presence of methanol, high temperatures, and acidic medium. Among the tested feedstocks, the enzyme showed the highest preference for macaw palm oil, producing a hydrolyzate with a final free fatty acid content of 92%. Crystallographic studies revealed a closed conformation of the helical amphipathic *lid* that typically undergoes conformational changes in a mechanism of interfacial activation. Such conformation of the *lid* is stabilized by a salt bridge, not observed in other structurally characterized homologs, which is likely involved in the tolerance to organic solvents. Moreover, the lack of conservation of the aromatic cluster IxxWxxxxxF in the *lid* of *Re*Lip with the natural mutation of the phenylalanine by an alanine might be correlated with the preference of short acyl chains, although preserving catalytic activity on insoluble substrates. In addition, the presence of five acidic amino acids in the *lid* of *Re*Lip, a rare property reported in other lipases, may have contributed to its ability to tolerate and be effective in acidic environments. Therefore, our work describes a new fungal biocatalyst capable of efficiently hydrolyzing macaw oil, an attractive feedstock for the production of “drop-in” biofuels, with high desirable feature for industrial conditions such as thermal and methanol tolerance, and optimum acidic pH. Moreover, the crystallographic structure was elucidated, providing a structural basis for the enzyme substrate preference and tolerance to organic solvents.

## Introduction

The increasing demand for energy that complies with the current restrictions on emissions of polluting gases is promoting the development and production of alternative fuels from sustainable and renewable sources (Yan et al., 2015; Wang et al., 2018). The biohydrocarbons, in particular, medium- and long-chain fatty alkanes and alkenes, represent very promising alternatives for replacing the fossil fuels used in the transportation sector, once they have similar chemical composition and physical characteristics to those of conventional fuels derived from petroleum (Xu et al., 2017). Therefore, biohydrocarbons can share the infrastructure for storage and transportation already used for the distribution of gasoline, diesel, and jet fuel (Yan et al., 2015; Sousa et al., 2018) and can be combined at any proportion with those fuels without any modification in the engines and other mechanical components of the vehicles (Sousa et al., 2018).

Macaw palm (*Acrocomia aculeate*) is a native species distributed in the tropical and subtropical Americas, and it occurs naturally from southern Mexico to northern Argentina (Ciconini et al., 2013; Pires et al., 2013; Silva et al., 2014; da Conceição et al., 2017). It is predicted that a commercial plantation under appropriate agronomic conditions can produce from 16,000 to 25,000 kg of fruit per hectare (Pires et al., 2013). Its fruits are oil rich, accumulating up to 70% of oil (dry weight) and yielding approximately 6,200 kg of oil per hectare (Pires et al., 2013; Silva et al., 2014). The macaw palm has interesting agronomic and ecological features because it can occupy degraded areas or agroforestry systems given that it has high plasticity to grow in different ecosystems (Moura et al., 2009; Pires et al., 2013), avoiding conflict with areas used for food production. Macaw pulp oil presents a high content of free fatty acids (FFA) and, thus, elevated acidity (Lopes et al., 2013), with oleic acid (C18:1) being the predominant fatty acid (Bora and Rocha, 2004). Thus, it can generate a high-quality biofuel, with high amounts of monounsaturated compounds (Aguieiras et al., 2014).

Currently, the chemical hydrotreatment of acyl glycerides, fatty acids, or esters is the route commonly applied to obtain biohydrocarbons (Gosselink et al., 2013; Kim et al., 2014; Chen et al., 2016; Karatzos et al., 2017; Pattanaik and Misra, 2017; Khan et al., 2019; Scaldaferri and Pasa, 2019). However, this process requires high-cost metallic catalysts, large amounts of hydrogen, elevated temperatures and high-pressure conditions, which makes it not economically viable, technologically complex, and environmentally unfavorable (Zhang et al., 2011; Yan et al., 2015; Sousa et al., 2018; Li et al., 2019). Alternatively, enzymatic routes for biohydrocarbon production have been investigated since they can act at mild industrial conditions and present high selectivity, which precludes undesirable side reactions, besides providing pure products with high yields (Beller et al., 2010; Schirmer et al., 2010; Wang and Lu, 2013; Herman and Zhang, 2016; Scrutton, 2017; Wise et al., 2017; Zargar et al., 2017; Knoot and Pakrasi, 2019).

Lipases (triacylglycerol acylhydrolases, E.C. 3.1.1.3) are water-soluble enzymes (Reis et al., 2009) that can catalyze oil hydrolysis, esterification, and transesterification (Goswami et al., 2013). Most of them are interfacial enzymes, which means that, at the oil–water interface, they undergo structural changes that involve a movement of a short helical segment (*lid*) that covers the active site. Thus, during the activation process, the *lid* changes its conformation, exposing the catalytic triad and increasing the aliphatic surface surrounding the active site, required for substrate binding (Derewenda et al., 1992; Maruyama et al., 2000; Gruber and Pleiss, 2012). In addition, due to their transesterification activity, during the last decade, lipases have been extensively studied as a promising industrial biocatalyst for biodiesel production (Bajaj et al., 2010; Hwang et al., 2014; Norjannah et al., 2016; Amini et al., 2017). It has recently been reported that few vegetable lipases, especially those from castor bean seeds, are more effective on the hydrolysis of macaw oil because they usually act at acidic pHs compared to fungal lipases that perform under more basic conditions (Ory et al., 1962; Muto and Beevers, 1974; Avelar et al., 2013; Bressani et al., 2015).

Therefore, considering the interest to make macaw palm an alternative platform to produce renewable biofuel and other bioproducts, and the very limited knowledge of biocatalysts available that can convert this specific acidic oil, the aim of this work was to develop a fungal lipase that could be compatible and efficient for this feedstock. *Rasamsonia emersonii* is an important microbial platform for cellulose degradation since its genome possesses many effective glycoside hydrolases (Gudmundsson et al., 2016; Martínez et al., 2016); nevertheless, it remains underexplored regarding enzymes active on triglycerides. For the first time, we have cloned and heterologously expressed the lipase from *Rasamsonia emersonii* (*Re*Lip). The functional analysis revealed that *Re*Lip has an optimum acidic pH profile and was active on a wide range of substrates from short to long carbon length chains. The enzyme was remarkably active on macaw oil, being able to produce a total of 92% FFA content. Furthermore, *Re*Lip is highly thermo-tolerant under extreme conditions such as in the presence of methanol. Crystallographic analysis revealed the closed conformation of this lipase indicating structural properties driving substrate selectivity and tolerance to organic solvents. In this sense, this work, besides providing a novel effective biocatalyst suitable for typical harsh industrial conditions, reveals new molecular aspects associated with substrate preference and tolerance to extreme conditions.

## Materials and Methods

The lipase gene from *Rasamsonia emersonii* cloned into the pET28a(+) vector with a histidine-tag at the N-terminus was purchased from Genscript (Piscataway, NJ, United States) (GenBank accession number: 915129218). Gum Arabic and ethanol (minimum 99.5%) were purchased from Synth and acetonitrile (minimum 99.9%) from Tedia. Commercial soybean (Liza), canola (Liza), olive (Gallo), sunflower (Liza), corn (Liza), and coconut oils were obtained at local markets. Palm and macaw oils were kindly supplied by Agropalma and Dr. Carlos Colombo [Agronomic Institute of Campinas (IAC), respectively]. FFA composition and percentage of FFA (acidity) of the vegetable oils used in this work are described in Supplementary Table S1. The FFA content was measured according to AOCS standard number Ca 5a-40 (Walker, 1990). The fatty acid composition of each oil was determined using a Pegasus HT (Leco) connected to a gas chromatographer (7890A, Agilent), according AOCS standard Ce 1-62 method (AOCS, 2005).

### Protein Expression and Purification

The plasmid *Re*Lip1-pET28a was produced in *E. coli* BL21(DE3)pLysS strain with pRARE2 plasmid. The cells were cultured in Luria–Bertani (LB) agar containing 25 μg/ml of kanamycin and chloramphenicol. For growth, one colony was picked and cultured in 5 ml of LB broth containing 25 μg/ml of kanamycin and chloramphenicol, with overnight shaking at 37°C and 250 rpm. After this period, the material was diluted 100-fold with fresh selective terrific broth (TB) and incubated at 37°C until an optical density (OD_600__*nm*_) of 1.2. Thus, the protein expression was subsequently induced by 0.5 mM isopropyl β-D-1-thiogalactopyroanoside (IPTG) and cultivated for 16 h at 20°C and 250 rpm. The cells were collected by centrifugation at 8,000 × *g* for 20 min at 4°C and resuspended in 20 ml of lysis buffer (50 mM phosphate pH 7.4, 300 mM NaCl, 30 mM imidazole, 1 mM phenylmethylsulfonyl fluoride (PMSF), and 0.5 mg ml^–1^ of lysozyme). The cells were, then, lysed by sonication, and the soluble protein was extracted by centrifugation at 12,000 × *g* for 30 min at 4°C. Thereafter, the enzyme was purified from the supernatant by two chromatographic steps: (i) metal-affinity chromatography, using a non-linear imidazole gradient (20–500 mM) in a 5-ml HisTrap HP column (GE Healthcare), previously equilibrated with 50 mM phosphate buffer, pH 7.4, 20 mM imidazole, and 300 mM NaCl and (ii) size-exclusion chromatography using a Superdex 200 HiLoad 16/60 column (GE Healthcare), previously equilibrated with 50 mM phosphate buffer, pH 7.4 and 150 mM NaCl coupled to an AKTÄ Purifier FPLC system (GE Healthcare Life Sciences). All steps of heterologous expression and enzyme purification were analyzed by sodium dodecyl sulfate-polyacrylamide gel (12% polyacrylamide) electrophoresis (SDS-PAGE). The protein concentration was spectroscopically determined using the molar extinction coefficient calculated from the amino acid composition^1^, which is 38,765 M^–1^ cm^–1^.

### Biochemical Characterization on Synthetic Substrates

The substrate specificity of *Re*Lip was evaluated at the following substrates: *p*NP palmitate (C16:0), *p*NP myristate (C14:0), *p*NP dodecanoate (C12:0), *p*NP decanoate (C10:0), *p*NP octanoate (C8:0), *p*NP valerate (C5:0), *p*NP butyrate (C4:0), *p*NP acetate (C2:0), and *p*NP format (C1:0). All *p*NP solutions (20 mM) were prepared in acetonitrile/isopropanol (1/4 *v/v*) and 0.3% (*v/v*) Triton X-100. The experiments were carried out in 96-well plates, with a reaction volume of 100 μl composed of 0.5 mg of purified enzyme, 40 mM citrate buffer (pH 3.5), and 5.0 mM *p*NPs. The system was incubated for 20 min at 65°C in a Veriti Thermal Cycler (Applied Biosystems, United States), and the reactions were ended by adding 100 μl of pure acetonitrile. The final absorbances were measured at 348 nm, which is the isosbestic point of *p*-nitrophenol and *p*-nitrophenoxide (Rhee et al., 2005; Glogauer et al., 2011), using an Infinite^®^200 PRO microplate reader (TECAN Group Ltd., Switzerland). All reactions were performed in quadruplicates. Control points were made using water instead of enzyme. The measurements were expressed as relative activity (%) considering the maximum catalytic activity observed for the biological unit of the enzyme.

The optimum temperature and pH of the enzyme was determined spectrophotometrically following the hydrolysis of *p*-nitrophenylbutyrate (*p*NPB; C4:0; Sigma-Aldrich Co., St. Louis, United States) at 348 nm. The reactions were performed as described above. The thermostability and the optimal temperature of the *Re*Lip were evaluated in a temperature range of 35°C to 75°C, at pH 4.0. The optimum pH for *Re*Lip activity was determined in the pH range of 2.0 to 6.0 at 65°C, using the following buffers: HCl-glycine (pH 2.0 and 2.5) and citrate (pH 3.0, 3.5, 4.0, 4.5, 5.0, 5.5, and 6.0), at a final concentration of 40 mM.

The additives tested were cations (NaCl, CaCl_2_, MgCl_2_, KCl, BaCl_2_, MnCl_2_, NiCl_2_, CoCl_2_, CuCl_2_, and FeCl_3_ at 10 mM), anions (H_2_SO_4_, CH_3_COOH, H_3_PO_4_, and HNO_3_ at 10 mM), detergents (Tween 20, Tween 80 and sodium docecyl sulfate, SDS (1%) and Triton X-100 from 0.1 to 10%), chelating agent (ethylenediamine tetraacetic acid, EDTA at 10 mM), modifying agents (diethyl pyrocarbonate, DEPC at 1 mM), and gum arabic, from 0.1% to 10% (v/v).

Each additive was investigated in individual 100-μl reactions containing 5 mM substrate *p*NPB, 100 mM citrate–phosphate buffer (pH 3.5), and 0.5 g/L of enzyme. The samples were incubated at 65°C for 20 min, and then, 100 μl of pure acetonitrile was added to stop the reaction. The activity was determined by absorbance measurement at 348 nm and compared to the control, which was incubated without any compound. The activity measured without additives was defined as 100%. Then, the evaluation was expressed as relative activity (%).

### Circular Dichroism (CD) Analysis

Circular dichroism (CD) measurements were carried out in a Jasco J-815 spectropolarimeter (Jasco, United States), using a Peltier-type temperature control system for temperature maintenance inside the cell. For the analysis of the secondary structure, the purified and homogeneous enzyme was diluted to multiple buffers in different conditions. Data were collected from 260 to 190 nm at 20°C, using a N_2_ flow rate of 10 ml/min, quartz cuvette with 0.1 cm of path length, scan speed of 50 nm/min, response time of 1.0 s, spectral bandwidth of 1.0 nm and spectral resolution of 0.1 nm. The final CD spectrum obtained was an average of 20 accumulations.

The CD spectroscopy was also applied to determine the protein stability at denaturing conditions, by measuring thermal-induced unfolding and melting temperature. For this purpose, the sample was heated from 20°C to 100°C at a rate of 1.0°C/min. The reversibility of the temperature effect was evaluated by cooling the denatured sample from 100°C to 20°C, using the same parameters described above. The values obtained in CD measurements (mDeg) were normalized to residual molar ellipticity (MRE), and the temperature at the midpoint transition (*T*_*m*_) was obtained by fitting the CD data to a sigmoidal Boltzmann function. Data were treated using the software Origin 8.1 (OriginLAB Corporation).

### Determination of Hydrolytic Activity Using Triacylglycerol Substrates

The hydrolytic activity of the *Re*Lip was determined on the hydrolysis of emulsified vegetable oils (Soares et al., 1999), aiming at verifying the optimum temperature and pH using triacylglycerol substrates and assessing the substrate specificity of the lipase. For the substrate specificity tests, vegetable oils such as soybean, canola, olive, sunflower, palm, macaw, corn, and coconut were used. The substrates were prepared by the emulsion of 7.5 g of vegetable oil, 67.5 g of water, 6.57 g of gum arabic (10% of water weight), and 60 ml of citrate buffer (pH 4.0, 50 mM), resulting in a final oil concentration of 5%. For the experiments, protein (at a final concentration of 2 g/L) and substrate were added (final reaction volume of 10 ml) into 125-ml Erlenmeyer flasks and incubated at 60°C and 200 rpm for 5 min in an incubator shaker (Innova 44, New Brunswick Scientific, United States). After the incubation period, the reactions were ended by the addition of 10 ml of ethanol P.A. (Synth).

For the analysis of optimum temperature and pH and the design of experiments, the hydrolysis reactions were performed using pulp macaw oil. The emulsion was prepared as described above. Thus, substrate and protein, at pre-defined proportions and final volume of 10 ml, were added into 125-ml Erlenmeyer flasks and incubated at 35°C and 200 rpm. After the desired time of reaction, the experiments were stopped by the addition of 10 ml of acetonitrile. The fatty acids produced in the hydrolysis were titrated with KOH solution, 0.2 mol L^–1^, using phenolphthalein as indicator. The hydrolysis degree was calculated according to Eq. (1) (Rooney and Weatherley, 2001):

(1) Hydrolysisdegree(%)=(V⁢x⁢ 10-3⁢x⁢MKOH⁢x⁢MMFFAw⁢x⁢f)x 100

where *V* is the volume of potassium hydroxide solution required in the titration process (ml), *M*_KOH_ is the KOH solution concentration (mol/L), *MM*_*FFA*_ is the average molecular mass of fatty acids of the macaw oil (276.01 g/mol), w is the weight of the sample titrated (g), and f is the fraction of oil in the begging of the reaction.

### Design of Experiments and Optimization

The experiments were performed at 35°C and pH 4.0. A matrix of 12 experiments were performed in duplicate, using alpha for orthogonality (α) of 1.21 and four replications at the center point. The independent parameters were defined as follows: catalyst concentration (*C*) and time of reaction (*t*). Supplementary Table S2 shows the variables used in the central composite design (CCD), with their five coded and uncoded levels. The response variable was defined as the hydrolysis degree. The hydrolysis degree obtained were fitted in a quadratic model using regression analysis, and for selecting the significant terms of the model, parameters with values of *p* < 0.05 were considered statistically significant. The range of each parameter was selected according to preliminary tests. For the optimization process, canonical analysis technique was employed (Box and Tiao, 1977). Thus, aiming at validating the optimization, the enzymatic hydrolysis was performed under the optimal experimental conditions suggested by the analysis. In this work, the CCD and RSM were developed by Statistica software version 7.0 (Statsoft, United States), and the canonical analysis was implemented using the software Maple 17.

### Dynamic Light Scattering (DLS)

Dynamic light scattering (DLS) technique was used to determine the hydrodynamic behavior of *Re*Lip in buffer purification at different concentrations (1–10 mg/ml). The experiments were carried out at room temperature using a Malvern Zetasizer Nano ZS90 (Malvern Instruments, Worcestershire, United Kingdom) with a 633-nm laser, in a quartz cell with a scattering angle of 90°. The diffusion coefficient (DT) was determined from the analysis of measured time-dependent fluctuations in the scattering intensity and used to calculate the hydrodynamic radius (Rh) of the protein according to the Stokes–Einstein equation.

### Small-Angle X-Ray Scattering (SAXS)

Small-angle X-ray scattering measurements were acquired using a monochromatic X-ray beam (λ = 1.488 Å) from the D01A-SAXS2 beamline at the Brazilian Synchrotron Light Laboratory (LNLS, Brazil). *Re*Lip (2–4 mg/ml) samples were prepared in buffer purification. Prior to conducting the SAXS experiments, all samples were centrifuged for 15 min at 20,000 × *g* and 4°C to remove any potential residual aggregates. The sample-to-detector distance was set as 1,000 mm, resulting in a scattering vector (*q*) range of 0.02 Å^–1^ < *q* < 0.50 Å^–1^, where the *q*-vector magnitude is defined as *q* = 4πsinθ/λ in which 2θ is the scattering angle. Samples were analyzed at 20°C and placed in 1-mm-path-length mica cells, and the scattering profiles were recorded in 10 successive frames (30 s each) to monitor radiation damage. The buffer contribution in each SAXS profile was subtracted taking into account the attenuation and integrated in the sample using the FIT2D software (Hammersley, 2016). Sample mono dispersity was checked by means of the *Guinier’s law*, and all studied systems presented here were found to be monodisperse, and no aggregation took place over the SAXS curves (data not shown). The *Gnom* program (Svergun et al., 1988) was also used to generate the pair distance distribution function [*p*(*r*)] and the protein maximum dimension (*D*_*max*_) from scattering profiles. Employing the *p*(*r*) function, the *DAMMIN* software (Svergun, 1999) was applied to obtain *ab initio* models for *Re*Lip (dummy atom model) by a simulated annealing optimization routine that yields a best fit to the experimental scattering data. Shapes were reconstructed by averaging a minimum of 10 different *ab initio* models using the *DAMAVER* package (Volkov and Svergun, 1995). The experimentally derived low-resolution envelopes were superimposed on structures obtained by molecular modeling using the *SUPCOMB* software (Kozin and Svergun, 2001).

### Crystallization, Data Collection, and Processing

Multiple amino acid sequence alignment of fungal lipase sequences was performed using Clustal Omega and ESPript 3.0. The following lipases were aligned: 1DT3: *Thermomyces lanuginosus* lipase, 62.08% identity; 1TIA: *Penicillium camemberti* lipase, 49.26% identity; 5CH8: *Penicillium cyclopiu*m lipase, 48.90% identity; 5XK2: *Aspergillus oryzae* lipase, 42.35% identity; 3NGM: *Fusarium graminearum* lipase, 45.76% identity; 4L3W: *Rhizopus microsporus var. chinensis* lipase, 29.92% identity; 1LGY: *Rhizopus Niveus* lipase, 31.65% identity; 5TGL: *Rhizomucor miehei* lipase, 34.53% identity; 2HL6: *Aspergillus niger feruloyl* esterase, 30.85% identity (Supplementary Figure S1). Protein sample at a concentration of 16.5 mg ml^–1^ in 50 mM sodium phosphate, pH 7.4, and 150 mM NaCl buffer was used in the crystallization experiments. The sample was under sitting-drops prepared at 18°C using a Cartesian HoneyBee 963 system (Genomic Solutions). Conditions (544) were tested, based on commercially available crystallization kits from Hamptom Research (SaltRx, Crystal Screens I and II), Emerald BioSystems (Precipitant Synergy and Wizard I and II), and Qiagen/Nextal (PACT and JCSG+). The drop, composed of 0.7 μl of the protein solution and 0.3 μl of the condition, was equilibrated over the reservoir containing 80 μl of the respective solution. Crystal optimization was performed using a systematic grid in which PEG 8000 concentration (from 20% to 8%) was varied in function of PEG 400 (from 20% to 8%) in a solution of 100 mmol/L MgCl_2_ and 100 mmol/L Tris buffer pH 8.5. Two crystals with approximate dimensions of 30 × 30 μm were obtained. The solution consisted of 100 mmol/L MgCl_2_, 20% PEG 8000, 20% PEG 400, 100 mmol/L Tris buffer pH 8.5. The crystallization conditions are summarized in Supplementary Table S3.

Diffraction data were collected at the BL12-2 beamline from the Stanford Synchrotron Radiation Lightsource (Menlo Park, CA, United States). Crystals were cooled in liquid nitrogen and kept under a nitrogen gas stream during the collection at 100 K. A total of 1,800 images were collected, and data were indexed, integrated, and scaled using XDS package (Kabsch, 2010). Pointless (Evans, 2006, 2011) and Zanuda (Lebedev and Isupov, 2014) were used to select and validate the chosen space group. Data analysis was performed with Xtriage (Zwart et al., 2005; Adams et al., 2010), and detection of anisotropy was made using the diffraction anisotropy server (Strong et al., 2006). Supplementary Table S4 presents the parameters used in the data processing.

## Results and Discussion

### *Re*Lip Presents an Uncommon Acidic pH-Activity Profile and Its Catalytic Activity Is Not Metal Dependent

*Re*Lip was overexpressed in the soluble and stable form in *E. coli* BL21(DE3)plysS yielding 120 mg/L of TB medium. After steps of purification, fractioned samples were analyzed by SDS-PAGE (Supplementary Figure S2). *Re*Lip showed a single band between 31 and 45 kDa after size exclusion chromatography, corresponding to the expected molecular weight of the construct with an N-terminal His-tag (31.75 kDa).

**FIGURE 1:**
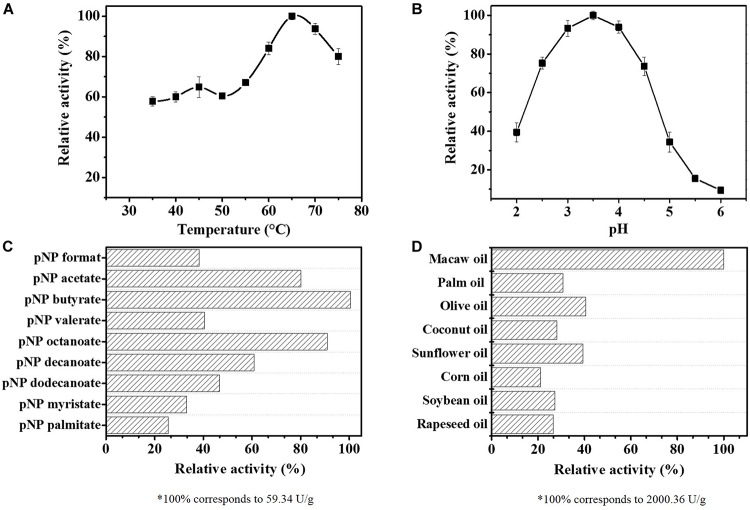
Effect of temperature **(A)** and pH **(B)** on the activity of the purified lipase from *Rasamsonia emersonii* (*Re*Lip). The temperature effect was evaluated from 35°C to 75°C, at pH 4.0, while pH influence was assessed at 65°C using different buffers (pH 2.0 to 6.0). **(C)** *Re*Lip specificity against synthetic substrates (*p*NPs) with different chain lengths (65°C, pH 3.5, incubated for 20 min); 100% of activity corresponds to 59.34 U/g. **(D)** Hydrolytic activity of *Re*Lip using different vegetable oils (5 wt%, 60°C, pH 4.0, incubated for 5 min); 100% of activity corresponds to 2,000.36 U/g. All measurements were conducted in triplicate. The relative activity (%) was calculated considering the maximum catalytic activity observed for the biological unit of the enzyme, and the specific activity was defined as the number of units per g of protein.

The effects of pH and temperature on *Re*Lip activity were investigated using *p*NPB as substrate. Figure 1A shows the effect of temperature variation on the enzymatic activity, from 35°C to 75°C at pH 4.0. The optimum temperature profile revealed that *R*eLip possesses a high thermal stability, with a relative activity of 60% at lower temperatures and a maximum activity around 65°C when analyzed using a synthetic substrate. In Figure 1B, it is possible to observe the effect of pH, varying from 2 to 6. According to the pH profile, *Re*Lip works better at acidic conditions, with an optimum pH around 3.5. Based on these results, *Re*Lip shows an interesting feature when compared to most of the industrial lipases from fungi. Usually, lipases, mainly from microbial origin, act in an alkaline spectrum (Papaparaskevas et al., 1992; Soares et al., 1999; Hiol et al., 2000; Saxena et al., 2003; Yu et al., 2007; Utsugi et al., 2009; Belhaj et al., 2011). Only a few lipases, like the lipase from *Aspergillus niger*, show such acidic profile (Romero et al., 2007).

Knowing that additives can improve the enzymatic activity of lipases (Priyanka et al., 2019), *Re*Lip was tested in the presence of a wide range of additives, such as detergents, chelating and modifying agents, cations, anions, and gum arabic. The results are shown in Table 1. Among the salts tested, only NaCl increased the relative activity. Considering this result, an investigation using different concentrations of NaCl was carried out, aiming at better understanding the effect of ionic strength on enzymatic activity. In the presence of 50 mM NaCl, *Re*Lip had its relative activity increased twofold with small increments in the activity up to 0.5 M. Inhibition effects were observed only at concentrations higher than 1 M. Calcium was also evaluated in the range from 0.01 to 2 M, since this divalent cation is usually an important cofactor for lipase activity (Shibata et al., 1998; Simons et al., 1999; Choi et al., 2005; Tayyab et al., 2011; Lan et al., 2015). Different from typical lipases, no significant improvement in the activity was observed with calcium, indicating that *Re*Lip is not a metal-dependent enzyme. This was further supported by the fact that the chelating agent, EDTA, did not substantially affect the catalytic activity.

**TABLE 1:** Effect of additives on *Re*Lip activity.

Additives	Relative activity (%)	Additives	Relative activity (%)	Additives	Relative activity (%)

Cation (10 mM)		Sodium chloride		Calcium chloride	
Control	100.02.5	Control	100.03.9	Control	100.04.1
NaCl	142.24.5	0.010 M	155.011.2	0.010 M	121.52.7
CaCl_2_	122.84.7	0.050 M	203.39.2	0.05 M	92.73.9
MgCl_2_	107.28.5	0.10 M	151.53.5	0.10 M	90.55.6
KCl	101.17.1	0.15 M	145.36.8	0.15 M	74.88.1
BaCl_2_	90.64.5	0.20 M	122.07.6	0.20 M	40.34.3
MnCl_2_	85.75.3	0.50 M	102.54.9	0.50 M	39.68.6
NiCl_2_	77.46.3	1.0 M	86.18.2	1.0 M	34.67.7
CoCl_2_	66.36.8	1.5 M	50.01.5	1.5 M	23.63.8
CuCl_2_	53.54.1	2.0 M	37.57.1	2.0 M	23.37.0
FeCl_3_	29.17.0				

**Anion (10 mM)**		**Triton X-100**		**Gum arabic**	

Control	100.02.5	Control	100.04.7	Control	100.06.6
PO_4_^–^	68.71.6	0.10%	116.76.6	0.10%	112.03.1
SO_4_^–^	105.94.9	0.30%	137.511.8	0.30%	112.12.8
NO_3_^–^	62.92.2	0.50%	177.18.5	0.50%	112.31.5
CH_3_COO^–^	78.04.8	0.70%	173.97.9	0.70%	114.24.9
		1.0%	144.33.5	1.0%	133.31.9
		2.0%	138.73.4	2.0%	108.53.7
		4.0%	136.43.8	4.0%	107.23.6
		6.0%	115.910.6	6.0%	106.78.8
		8.0%	108.18.6	8.0%	111.92.4
		10.0%	95.512.2	10%	105.87.3

**Detergent (1%)**		**Chelating agent (10 mM)**		**Modifying agent (1 mM)**	
		
Control	100.02.5	Control	100.02.5	Control	100.02.5
Tween 20%	82.16.3	EDTA	80.75.7	DEPC	85.47.9
Tween 80%	79.55.4				
SDS	10.91.9				

The presence of gum arabic and detergents was also tested (Table 1), once those compounds are typically used in substrate emulsions to stabilize and improve emulsion quality (Glogauer et al., 2011). Regarding gum arabic, its presence did not considerably increase the activity or promote inhibition at any of the concentrations tested, indicating that this gum can be used in the preparation of substrate emulsions for the application of *Re*Lip. Among the detergents, SDS, Tween 80% and Tween 20% decreased the activity of the enzyme, especially SDS, with a remaining relative activity of only 11%. However, Triton X-100 showed a great and significant effect as additive for *Re*Lip activity. An increase in the concentration of this additive promoted an activation on the activity, achieving the highest relative activity of 177% at a concentration of 0.5%. The inhibition process only started at concentrations above 10%, possibly due to a denaturation process (Glogauer et al., 2011). Considering that Tween and Triton X-100 are non-ionic detergents (with low hydrophilic/lipophilic balance value), they probably did not interact extensively with the surface of proteins. On the other hand, ionic detergents, such as SDS, may have non-specific interactions with the surface of the proteins, facilitating structure denaturation (Priyanka et al., 2019).

### *Re*Lip Is a Lipase With Preference for Macaw Oil as Substrate

In order to identify the specificity of *Re*Lip, the enzymatic activity was evaluated as a function of different chain lengths of *p*-nitrophenyl substrates (Figure 1C). The results showed that *Re*Lip was able to hydrolyze all substrates tested. However, *Re*Lip preferentially hydrolyzed short acyl chains, showing higher activities against *p*NP butyrate (C4:0), *p*NP octanoate (C8:0), and *p*NP acetate (C2:0), with relative activities of 100%, 90.9%, and 80.1%, respectively. The lowest relative activity was observed against *p*NP palmitate (C16:0). It is known that one of the main differences between esterase and lipase is the fact that while esterase acts only on soluble substrates with smaller chains, lipases can hydrolyze insoluble substrates with larger chains above 10 carbons. The assays in the presence of *p*NPs with different sizes revealed that *Re*Lip has the ability to act on both short and long chains and consequently, *Re*Lip can be classified as an enzyme with esterase and lipase activity (Sharma et al., 2001, 2013; Jaeger et al., 2002; Silva et al., 2009). Thus, in order to prove the activity on insoluble substrates and confirm that *Re*Lip is a true lipase, the hydrolytic activity using different emulsified vegetable oils, such as soybean, canola, olive, sunflower, palm, macaw, corn, and coconut, was evaluated (Figure 1D). It is possible to observe that *Re*Lip was active against all oils tested, presenting a sequential preference for macaw oil (2,000 U/g), followed by olive oil (811 U/g), and sunflower oil (788 U/g). These results showed that the hydrolytic activity depends on a wide range of factors and not only on the length of the acyl chain and the amount of unsaturation. Probably, the high amounts of FFA in the macaw oil led to an acidification of the medium that benefited the hydrolytic activity of *Re*Lip.

**FIGURE 2:**
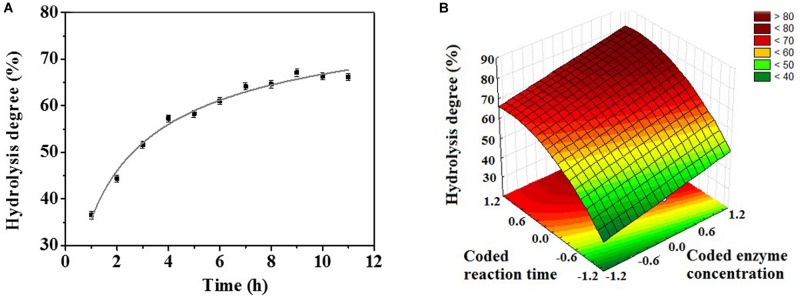
**(A)** Kinetic profile of macaw oil hydrolysis (5 wt%), at 35°C, pH 4.0, and enzyme concentration of 1 mg/ml. **(B)** Response surface for macaw oil hydrolysis degree as a function of coded reaction time (*X*_*t*_) and coded enzyme concentration (*X*_*C*_). Experimental conditions: 35°C and pH 4.0.

In view of the preference for hydrolyzing macaw oil, the optimum temperature and pH profiles using this oil as substrate were also evaluated (Supplementary Figure S3). The enzyme showed a distinct temperature dependence using the macaw oil as substrate compared to the synthetic substrate (*p*NPB), with an activity plateau in the temperature range from 25°C to 50°C (Supplementary Figure S3A). Despite that, the enzyme retained up to 73.3% of its activity after 24 h of incubation in this temperature range, indicating a high stability under these conditions (Supplementary Figure S3C). Regarding the pH profile, both synthetic and natural substrates exhibited the optimum pH at 4.0 (Supplementary Figure S3B). Thus, to obtain the kinetic profile of macaw oil hydrolysis, the experiments were set at 35°C and pH 4.0. The data revealed a hydrolysis degree of 50% with 3 h of reaction, and 63% was obtained with 8 h (Figure 2A). Consequently, a central composite design (CCD) coupled with response surface methodology (RSM) was performed in order to evaluate the effect of the experimental conditions “reaction time” and “enzyme concentration” on the enzymatic hydrolysis of macaw pulp oil. The enzyme concentration ranged from 0.5 to 1.8 mg/ml, according to preliminary tests. The range of reaction time was defined from 1 to 15 h, in which the center point was set at 8 h. Table 2 shows the experiments that were performed according to the CCD matrix, as well as the hydrolysis degree obtained for each experiment. The hydrolysis degree varied from 39% to 77%, in the evaluated range. The highest hydrolysis degree was obtained using 1.69 mg/ml and reaction time of 13:47 h (run 4). With the experimental results, a quadratic regression model was fitted [Eq. (2)], by eliminating the parameters which were not significant (values of *p* > 0.05). Thus, the statistical model obtained [Eq. (2)] describes the effect of the significant parameters on the hydrolysis degree (*y*).

**TABLE 2:** Experimental design for the hydrolysis of macaw oil, with uncoded values of lipase concentration (*C*) and reaction time (*t*) and hydrolysis degree (%) obtained.

Run	Lipase concentration (mg/ml)	Reaction time (h)	Hydrolysis degree (%)
1	0.61	2 h 12 min	48.25 ± 0.26
2	0.61	13 h 47 min	66.80 ± 0.03
3	1.69	2 h 12 min	58.34 ± 0.06
4	1.69	13 h 47 min	77.35 ± 1.14
5	0.50	8 h	57.65 ± 1.38
6	1.80	8 h	72.68 ± 0.15
7	1.15	1 h	39.06 ± 0.33
8	1.15	15 h	76.55 ± 0.76
9	1.15	8 h	68.77 ± 1.16
10	1.15	8 h	69.11 ± 0.79
11	1.15	8 h	68.13 ± 0.67
12	1.15	8 h	68.30 ± 1.00

*Experimental conditions: 35°C and pH 4.0.*

(2) $$y\left(\%\right)=67.63+5.60X_{C}+11.97X_{t}-5.86X_{C}^{2}$$

where *X*_*C*_ and *X*_*t*_ correspond to coded values for lipase concentration and reaction time, respectively. Eq. (2) had a correlation coefficient *R*^2^ of 0.9393 and adjusted *R*^2^ of 0.9166, which means that the statistical model accurately describes the correlation between the actual and predicted responses (Jun et al., 2019). The analysis of variance (ANOVA) for the model is presented in the Supplementary Material Section (Supplementary Table S5).

The regression coefficients of Eq. (2) show that reaction time (*X*_*t*_) was the most significant parameter on the hydrolytic degree, with a coefficient of 11.97, followed by the quadratic effect of enzyme concentration ($X_{C}^{2}$), with a coefficient of 5.86, and, finally, the linear effect of enzyme concentration (*X*_*C*_), with 5.60. The interaction between the parameters was not significant. Furthermore, the signals of the regression coefficient show that all linear parameters positively affect the response of the process. In contrast, the quadratic term of enzyme concentration negatively affects hydrolytic activity.

Comparing the experiments in which *Re*Lip concentration was fixed and the time of reaction was changed (runs 1–2, 3–4, 7–8-center points), it is possible to observe the positive effect of reaction time on hydrolysis degree. Raising the variable from level −1 to level +1 promoted an increase of 1.35-fold in the hydrolysis degree. In addition, comparing runs where the time of reaction was fixed and enzyme concentration was changed (runs 1–3, 2–4, 5–6-center points), it is possible to observe that an increase in enzyme concentration also promoted an increase in the hydrolysis degree. However, this effect was less pronounced. From level −1 to level +1, an increase of 1.18-fold in the hydrolysis degree occurred. The effect of the independent variables on hydrolysis degree can also be observed at the 3D response surface plot (Figure 2B). It is possible to note that the region with the highest hydrolysis degree is obtained at the highest values of enzyme concentration and time of reaction, in accordance with the positive effect obtained in the statistic equation [Eq. (2)].

In order to maximize the hydrolysis degree, a canonical analysis for the complete regression model was applied. The canonical model is presented in Eq. (3). All characteristic roots (λ_1_ and λ_2_) presented negative signals, which means that a maximum stationary point was obtained. The optimal conditions suggested by the optimization were 3.0 mg/ml of enzyme (coded value 3.43) and 14 h 06 min of reaction (coded value 1.05), with a predicted hydrolysis degree of 84%. An experimental validation was conducted. The experiments at the optimal conditions were performed in duplicate. A hydrolysis degree of 81.47% was obtained, with a final FFA content of 92%, confirming the result predicted by the canonical analysis.

(3) $$y=84.04-5.86\,w_{1}^{2}-0.83\,w_{2}^{2}$$

The results obtained in the present work, which were hydrolysis degree of 81.5% and hydrolyzate with final FFA of 92.0%, are very promising, since *Re*Lip presented similar results on the hydrolysis of macaw pulp oil to those conducted with commercial and vegetable lipases (Table 3). As previously mentioned, macaw palm is a very productive oleaginous tree, adapted to various types of soil and to semiarid ecosystems (Moura et al., 2009), which avoids conflict with areas used for food production. Additionally, its acidic oil cannot be used as food (Aguieiras et al., 2014), making it a great alternative as feedstock for biofuel production.

**TABLE 3:** Comparison of final free fatty acid obtained from the hydrolysis of macaw pulp oil, using lipases from different sources.

Catalyst	Conditions of the hydrolysis reaction	Final free fatty acid of hydrolyzate (%)	References
Lipase from *Rasamsonia emersonii*	35°C, pH 4.0, oil concentration of 5% m/m, enzyme concentration of 3 mg/ml, 14 h of reaction	92%	This work
Enzyme extract from the castor bean	35°C, pH 4.5, 25% oil–water mass concentration and 288 U, 4 h of reaction	83%	Machado (2017)
Enzyme extract from dormant castor seeds	30°C, pH 4.0, oil concentration of 50% v/v, enzyme concentration of 2.5% m/v, 6 h of reaction	99.6%	Aguieiras et al. (2014)
Enzymatic extract from dormant castor bean seeds	35°C, pH 4.5, mass ratio oil:buffer of 35% m/m, enzyme concentration of 6% m/m, 110 min of reaction	100%	Bressani et al. (2015)
Commercial lipases: Lipozyme RM IM, Lipozyme TL IM, and Lipozyme 435.	55°C, pH 8.0, mass ratio oil:buffer of 2:1, Lipozyme RM IM concentration of 15% and 6 h of reaction	82%	Raspe et al. (2013)

### *Re*Lip Is Highly Thermo-Tolerant Under Acidic Conditions and in the Presence of Organic Solvents

**FIGURE 3:**
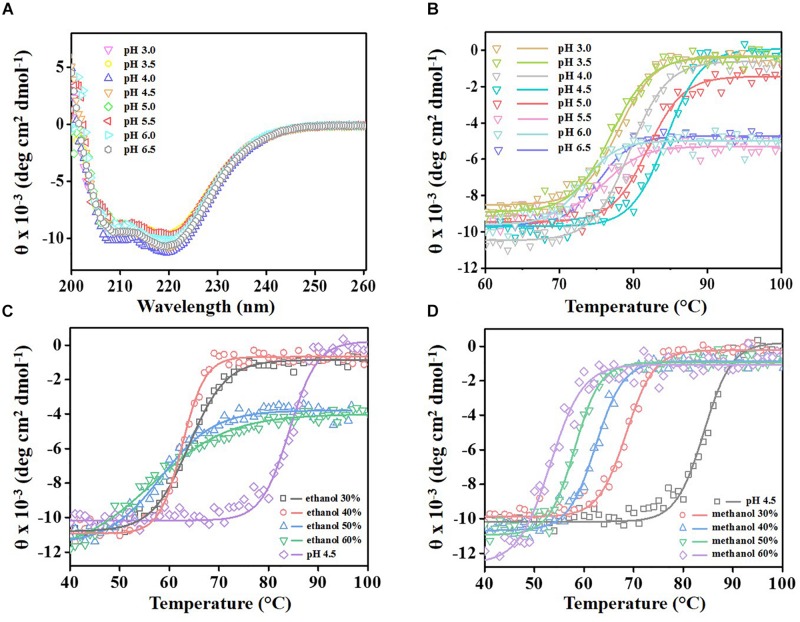
The far-UV averaged circular dichroism spectra of *Re*Lip, monitored at 20°C **(A)** at different pH conditions (from 3 to 6.5). **(B)** Thermal denaturation profile of *Re*Lip from 20°C to 100°C, monitored at 220 nm by circular dichroism spectroscopy at different pH conditions (from 3 to 6.5) and in the presence of different concentrations (from 30% to 60% v/v) of **(C)** ethanol and **(D)** methanol. The melting temperature (*T*_*m*_) varied from 73°C to 84°C for different pHs; maximum values were obtained at pH 4.5 and 5.0, with *T*_*m*_ of 84°C and 82°C, respectively. For ethanol: the melting temperature (*T*_*m*_) varied from 56°C to 64°C at different concentrations, in which the maximum value (64°C) was obtained with 30% of ethanol. For methanol: the melting temperature (*T*_*m*_) varied from 54°C to 68°C at different concentrations, in which the maximum value (68°C) was obtained with 30% of methanol.

To evaluate the conformational stability of *Re*Lip under harsh conditions such as low pH and the presence of organic solvents, the secondary structure profile was monitored by circular dichroism (CD) in a broad temperature range. CD analyses showed that *Re*Lip presents a typical α-helical profile, with minimum molar ellipticities at 208 and 222 nm. Furthermore, at different pH conditions (from 3 to 6.5), the secondary structure of the enzyme remained practically unchanged (Figure 3A). The analysis of the melting temperature (*T*_*m*_), by the measurement of thermal denaturation profiles at 220 nm (Figure 3B), revealed that the *T*_*m*_ varied from 73°C to 84°C for different pHs, with the maximum values obtained at pH 4.5 and 5.0, with a *T*_*m*_ of 84°C and 82°C, respectively. These results corroborate the data obtained previously, in which higher activities were observed under similar conditions. Thermo-tolerant lipases such as *Bacillus thermoamylovorans*, *Thermomyces lanuginosus*, and *Geobacillus thermoleovorans* have been reported in the literature, with thermal stability up to approximately 60°C (Sun et al., 2016; Mehta et al., 2017). *Re*Lip shows *T*_*m*_ values higher than other lipases considered thermostable, with an advantage of featuring high stability under acidic conditions, which is extremely desirable for many biotechnological applications, especially for biofuel production from crude or residual vegetable oils, which present high FFA contents.

The conformational changes in the secondary structures were also evaluated with *Re*Lip in the presence of organic solvents such as methanol and ethanol, compounds that are widely used in the biofuel industries. Surprisingly, *Re*Lip had its secondary structure unaffected in the presence of up to 60% ethanol and methanol at room temperature (Supplementary Figures S4A,B), which confirms its ability to tolerate organic solvents in high quantities, similar to that used in the industry. Figures 3C,D show the thermal denaturation profiles of *Re*Lip in the presence of these organic solvents. Changes in the enzyme thermal stability were observed; *T*_*m*_ values decreased as the amount of solvent was increased. In the presence of ethanol, from 30% to 60%, *T*_*m*_ varied from 64°C to 56°C. In the presence of methanol, it changed from 68°C to 54°C. Despite the decrease detected for both solvents, *T*_*m*_ values are significantly high, showing the good thermal stability of the enzyme and its high potential for application in reactions that involve those solvents. The phenomenon of *T*_*m*_ reduction in the presence of solvents may occur due to an alteration in the water-solvating layer (caused by organic solvents soluble in water) that surrounds the protein in aqueous solution, which may compromise the structural integrity of the enzyme. In addition, hydrophobic interactions of the structure can be affected by the presence of methanol and ethanol (Kamal et al., 2013; Sharma et al., 2016).

### *Re*Lip Has Unique Structural Properties That May Have Contributed to Its Acid and Methanol Tolerance

Despite a wealth of functional data on lipases, structural and mechanistic information are rather limited, especially for those from fungal origin. This is due to the difficulty of heterologous expression in a soluble and stable form, and the inherent recalcitrance to crystallization processes. The quaternary arrangement required for functional and structural stability is also partially understood with monomeric (*Rhizomucor miehei*) and dimeric (*Thermomyces lanuginosus*) lipolytic enzymes reported.

Consequently, the oligomeric arrangement of *Re*Lip was also assessed by DLS (Supplementary Figure S5A) and SAXS (Supplementary Figures S6A,B). DLS analysis revealed that *Re*Lip behaved as a monodisperse population (Pd < 15%) of monomers with an average hydrodynamic radius (Rh) of 3.05 nm. SAXS data showed that *Re*Lip has a gyration radius of 2.4 nm, which is in agreement with DLS data. In addition, the obtained envelope from the SAXS curve showed that the low-resolution structure of *Re*Lip consists of a monomeric globular form that corroborates with the three-dimensional structure obtained by X-ray crystallography (Supplementary Figure S6C).

**FIGURE 4:**
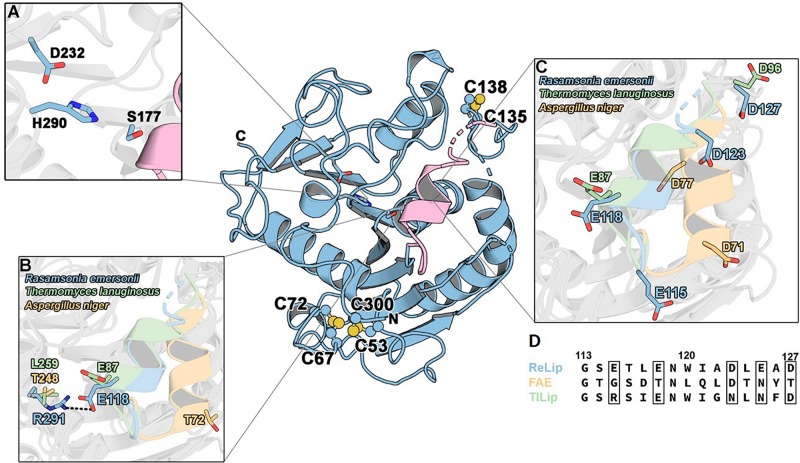
Tridimensional structure of *Re*Lip. **(A)** Catalytic triad: amino acids Ser177, His290, and Asp232. **(B)** Presence of a salt bridge formed between Arg291 and Glu118 from the *lid* subdomain. **(C)** *Lid* structures and **(D)** alignment between the primary sequences of the *lids* belonging to the lipases from *Re*Lip (this work), TLL (PDB code: 1DT3), and esterase from *Aspergillus niger* (PDB code: 1USW). The diffraction experiment was performed at SSRL-SLAC beamline BL12-2 with a wavelength of 0.98 Å. Crystal diffracted and collected at 2.4-Å resolution. The phases were obtained by molecular replacement using the three-dimensional structure of MDLA lipase from *Penicillium cyclopium* (PDB: 5CH8). The refinement was performed to the resolution of 3.0 Å (due to crystallographic anisotropy) using Phenix.

Aiming to get structural information regarding the interesting functional properties of *Re*Lip, the enzyme was submitted to crystallization tests. The obtained crystal (Supplementary Figure S5B) diffracted to 3.0 Å resolution, and the structure was solved using molecular replacement methods with the lipase from *Penicillium cyclopium* (PDB: 5CH8, 48.09% identity) as template. The *Re*Lip structure (PDB: 6UNV) presents an α/β hydrolase fold and consists of a major eight-stranded mixed β-sheet, two minor two-stranded β-sheet arrangements and five α-helices (Figure 4). Unlike its orthologs, *Re*Lip lacks the glycosylation site at position 33, which has a natural mutation with alanine instead of an asparagine. The literature reports that this site is important for the binding and recognition of micelles by TLL but apparently does not interfere in the catalytic activity (Peters et al., 2002). The lack of a glycosylation site in *Re*Lip structure may have contributed to its expression in a folded and stable form in a bacterial organism.

As expected, the catalytic triad of *Re*Lip consists of the residues Ser177, His290, and Asp232, which are fully conserved in other lipases and esterases (Figure 4A). *Re*Lip possesses three disulfide bonds (C67–C72, C53–C300, C135–C138) that are also conserved in TLL (Figure 4). One of them (C135–C138) is located close to the *lid* subdomain and is present in both lipases and esterases. The C53–C300 disulfide bridge is connecting the N- and C-termini, and is adjacent to the other C67–C72, conferring high structural stability to *Re*Lip.

Usually, the lipolytic activity of some lipases increases significantly at the critical micelle concentration of the substrate. This phenomenon of interfacial activation is related to the presence of a hydrophobic patch of the *lid* subdomain that is protected from the solvent adopting a closed conformation (Skjold-Jørgensen et al., 2014; Khan et al., 2017). With the recognition of the substrate, the *lid* undergoes conformational changes to expose the hydrophobic patch to interact with the substrate. However, in some lipases that have *minilid*s or do not even possess them, the interfacial activation does not occur, but these enzymes still present lipolytic activities. The three-dimensional structure of *Re*Lip (Figure 4C) confirmed the presence of a helical amphipathic *lid*, indicating that interfacial activation might be involved in *Re*Lip mechanism of substrate binding such as its ortholog TLL. Indeed, in the crystallographic closed conformation of *Re*Lip, the substrate could not be accommodated in the active-site cleft (Supplementary Figure S7), supporting that a conformational change is required for substrate binding. However, to better comprehend the molecular basis of the putative interfacial activation of *Re*Lip, an in-depth investigation is needed.

Lipases are also known by the occurrence of cluster IxxWxxxxxF, which confers a high hydrophobic character to the *lid* subdomain (Skjold-Jørgensen et al., 2014). The literature shows that the presence of the tryptophan residue is essential for the interfacial activation and substrate binding. *Re*Lip conserves the residues isoleucine and tryptophan, but the residue phenylalanine is replaced by an alanine. FAE also did not conserve the phenylalanine; instead, it has a tyrosine residue, which contributes to make its *lid* less hydrophobic (Hermoso et al., 2004). The lack of this aromatic residue in *Re*Lip might be associated with the preference for shorter acyl chains compared to TLL, while preserving the ability to cope with water-insoluble substrates.

Another singular feature of *Re*Lip is the presence of a salt bridge formed between Arg291 and Glu118 from the *lid* subdomain (Figure 4B). This salt bridge is not conserved in other structurally characterized lipases with the *lid* subdomain such as TLL. In TLL, the glutamate residue is conserved in the *lid*, but the arginine is substituted by a leucine. The presence of this salt bridge in *Re*Lip implicates in a higher protection of the hydrophobic pocket to bulky solvent, which can be correlated with the high tolerance of *Re*Lip to organic solvents. On the other hand, this salt bridge can represent an additional energetic cost for *Re*Lip to undergo the conformational changes required for substrate binding. The *lid* belonging to *Re*Lip also has a rare property when compared to other lipases and even esterases, which is the presence of five amino acids with acidic nature, which may have contributed to its robust performance under acidic conditions, thus favoring the hydrolysis of macaw oil, a substrate with high acidity.

**FIGURE 5:**
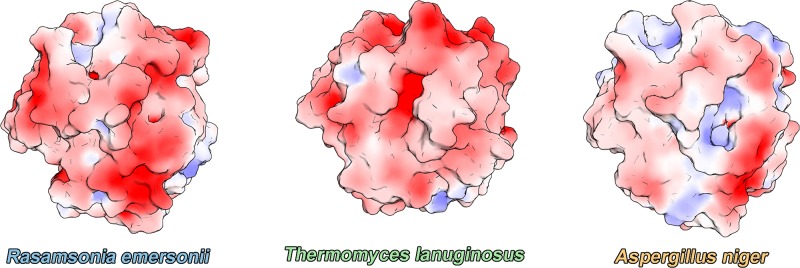
Electrostatic surface-potential of *Re*Lip (this work), TLL (PDB code: 1DT3), and esterase from *Aspergillus niger* (PDB code: 1USW), in which regions of negative and positive electrostatic potential are shown in red and blue, respectively.

Although esterases and lipases have a fully conserved catalytic site, charge distribution on the catalytic interface is reported to be quite unlike (Hermoso et al., 2004). While esterases exhibit a more neutral surface charge distribution, lipases have a negatively charged surface. Surface charge distribution has been associated with the better performance of FAE at lower pHs. However, the charge distribution of *Re*Lip largely differs from FAE, resembling that of TLL, despite having a better catalytic activity at lower pHs like FAE. This suggests that other factors aside from surface charge distribution at the catalytic interface are involved in the preference for acidic pHs for hydrolytic activity of these enzymes (Figure 5).

## Conclusion

Several lipases have been studied for the hydrolysis of vegetable oils; nevertheless, only a few, especially those from castor bean seeds, were capable of hydrolyzing macaw oil, an extremely acidic substrate coming from a native palm that is cultivated in a wide variety of soil types. In addition, the enzyme showed other desirable properties for vegetable oil processing, such as tolerance to organic solvents and high temperatures. The crystallographic structure was elucidated indicating the presence of a helical amphipathic *lid*, which might be involved in a mechanism of interfacial activation with the exposure of the hydrophobic patch for substrate binding. The typical aromatic cluster in the *lid* IxxWxxxxxF is not fully conserved in *Re*Lip with the natural mutation of the phenylalanine by an alanine. It decreases the hydrophobicity of the *lid* subdomain and is associated with the preference of short acyl chains, although preserving catalytic activity on insoluble substrates. The presence of a salt bridge between the *lid* and the vicinity of the active-site cleft confers a higher protection of the hydrophobic active site to bulky solvent, which might explain the tolerance of *Re*Lip to organic solvents.

## Data Availability Statement

The atomic coordinates and structure factors of *Re*Lip were deposited in the Protein Data Bank (http://ww.pdb.org/) under accession ID 6UNV. The original contributions presented in the study are included in the article/Supplementary  Material.

## Author Contributions

LR, MS, NM, and CS carried out the expression and purification of *Re*Lip and the oil hydrolysis reactions. RM and NM performed the functional experiments using synthetic substrates. NM performed the DLS analyses. MS carried out the CD experiments. PV, MM, and LZ designed and performed the crystallization trials, structure solution and refinement, and structural and SAXS analyses. LR, BK, HC, and AB contributed with the design of the experiments and with the analysis, treatment and discussion of the data obtained. LR wrote the article. LZ directed the overall study, analyzed the data, and wrote the manuscript. All authors read and approved the final manuscript.

## Conflict of Interest

The authors declare that the research was conducted in the absence of any commercial or financial relationships that could be construed as a potential conflict of interest.

## Footnotes

1 http://web.expasy.org/protparam/
