# Supplementary material for: A Novel Fungal Lipase With Methanol Tolerance and Preference for Macaw Palm Oil
Source: Front Bioeng Biotechnol. 2020 May 6;8:304. doi: 10.3389/fbioe.2020.00304 (PMC7218172; doi:10.3389/fbioe.2020.00304)
Supplement: Supplementary file 1 [file Data_Sheet_1.docx]

**Supplementary Material**

**A novel fungal lipase with methanol tolerance and preference for macaw palm oil**

**Letícia L. Rade^1^, Melque N. P. da Silva^1^, Plínio S. Vieira^1^, Natalia Milan^1,2^, Claudia M. de Souza^1^, Ricardo R. de Melo^1^, Bruno C. Klein^1^, Antonio Bonomi^1^, Heizir F. de Castro^2^, Mário T. Murakami^1^ and Leticia M. Zanphorlin^1^**

^1^ Brazilian Biorenewables National Laboratory (LNBR), Brazilian Center for Research in Energy and Materials (CNPEM), 13083-970, Campinas, São Paulo, Brazil

^2^ Department of Chemical Engineering, Engineering School of Lorena, University of São Paulo, 12602-810, Lorena, São Paulo, Brazil

***Correspondence:**

Dr. Leticia Zanphorlin

[leticia.zanphorlin@lnbr.cnpem.br](mailto:leticia.zanphorlin@lnbr.cnpem.br)

**
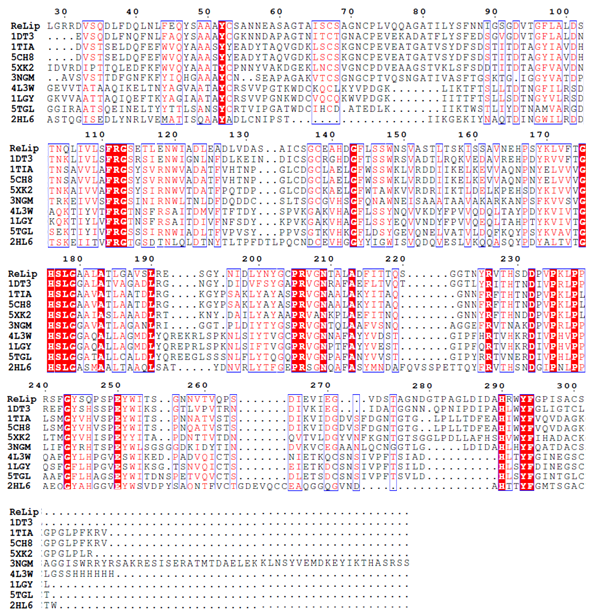
Figure S1**- Multiple amino acid sequence alignment of fungal lipase sequences using Clustal Omega and ESPript 3.0. PDB accession numbers of the aligned sequences for the following organisms: (**1DT3:** *Thermomyces lanuginosus* lipase, 62.08% identity; **1TIA:** *Penicillium camemberti* lipase, 49.26% identity; **5CH8:** *Penicillium cyclopium* lipase, 48.90% identity; **5XK2:** *Aspergillus oryzae* lipase, 42.35% identity; **3NGM:** *Fusarium graminearum* lipase, 45.76% identity; **4L3W:** *Rhizopus microsporus var. chinensis* lipase, 29.92% identity; **1LGY:** *Rhizopus Niveus* lipase, 31.65% identity; **5TGL:** *Rhizomucor miehei* lipase, 34.53% identity; **2HL6:** *Aspergillus niger* feruloyl esterase, 30.85% identity). High consensus regions are indicated inside red boxes and low consensus regions are in unfilled boxes. The numbers above the sequences represent amino acid positions.

**
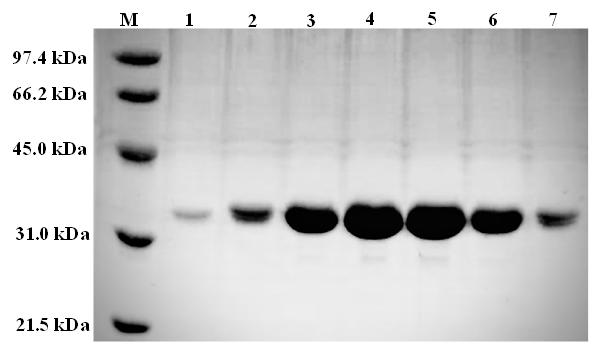
Figure S2** – Analysis of purified *Re*Lip (12% SDS-PAGE). Lane M, molecular weight marker; Lanes 1-7, *Re*Lip fractions obtained after molecular exclusion chromatography.

**(A)**

**(B)**

**(C)**

**Figure S3** – Functional analysis of *Re*Lip using macaw oil (5 wt%), 16 h of reaction and enzyme concentration of 0.6 mg/mL. (A) The effect of temperature on the hydrolysis degree, from 25 °C to 60 °C, at pH 4.0. (B) The effect of pH on the hydrolysis degree, from 3.0 to 6.0, at 35°C. The experiments were done in duplicate. (C) Thermal stability of *Re*Lip. Temperature stability was analyzed after pre-incubated pure *Re*Lip at 35 °C, 45 °C and 50 °C. The enzyme was incubated from 0 to 24 h and the hydrolysis reactions were conducted as described in Item 2.4.


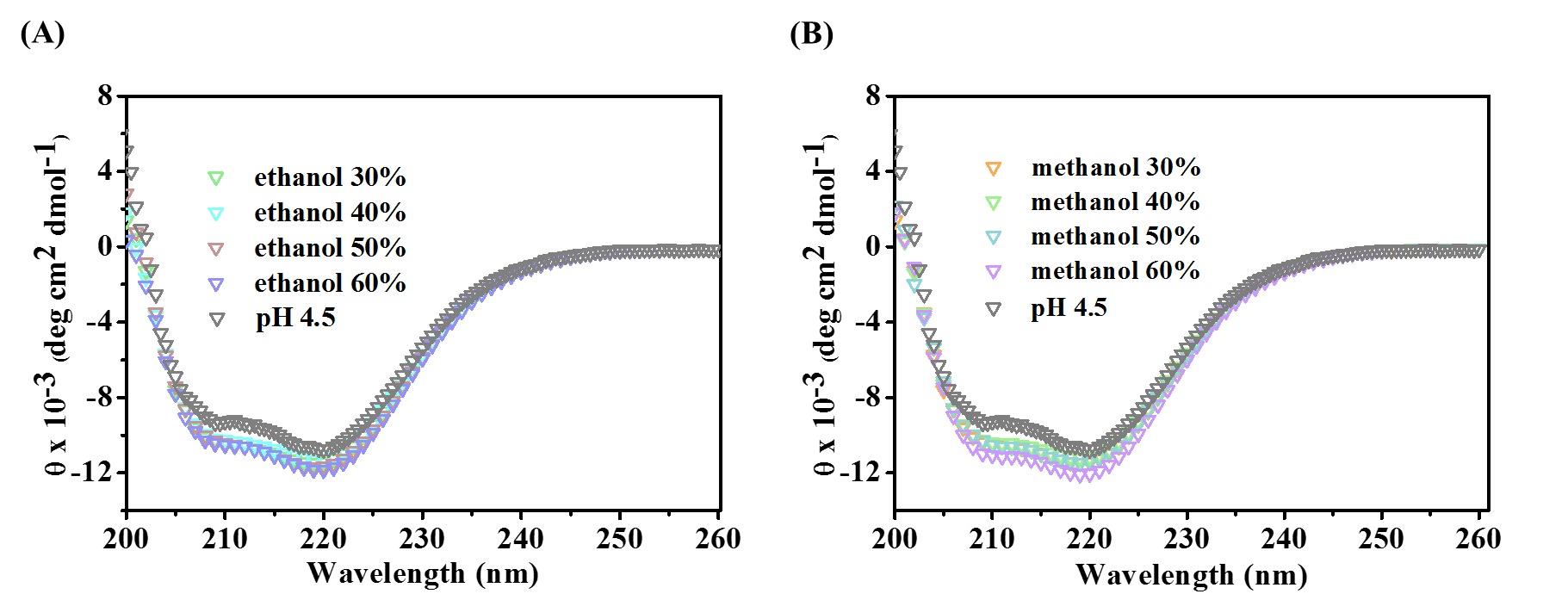


**Figure S4** – The far-UV averaged circular dichroism spectra of *Re*Lip, monitored at 20 ºC in different concentrations of (A) ethanol and (B) methanol.

**(B)**


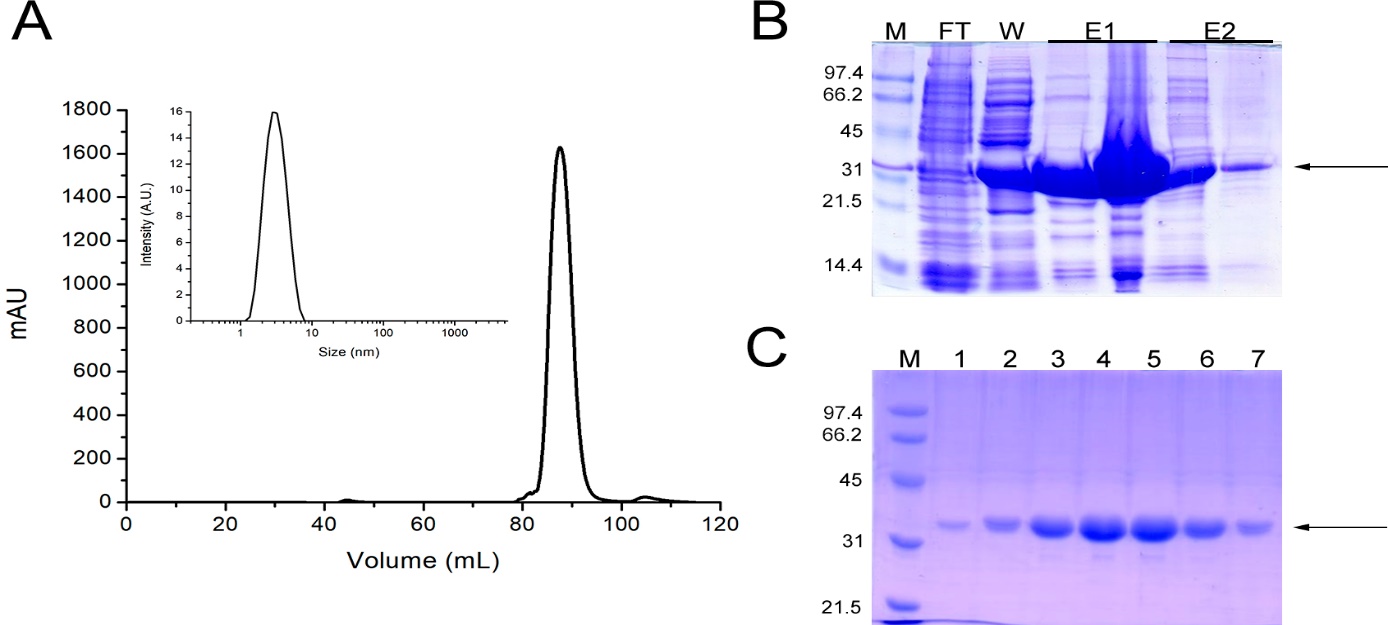


**(A)**


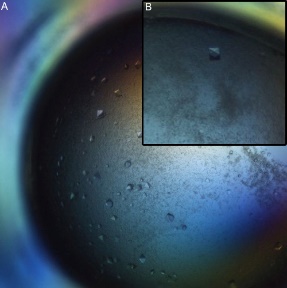


**Figure S5** – (A) Chromatogram obtained in the purification of *Re*Lip by size exclusion chromatography. The inset shows the hydrodynamic radius of the protein after the purification, obtained from DLS experiments, with a single peak at approximately 3 nm. (B) Single crystal obtained (30 x 30 µm). The purified protein was crystalized by sitting drop method at 18 °C, using the following condition: 100 mmol.L^-1^ MgCl_2_, 20% PEG 8000, 20% PEG 400, 100 mmol.L^-1^ Tris pH 8.5.

**Figure S6** – (A) Small Angle X-ray Scattering (SAXS) measurements of *Re*Lip. (B) Distance distribution function P(r). (C) SAXS ab initio envelopes with the crystallographic structure fitted, confirming the monomeric globular form that corroborates the three-dimensional structure obtained by X-ray crystallography.


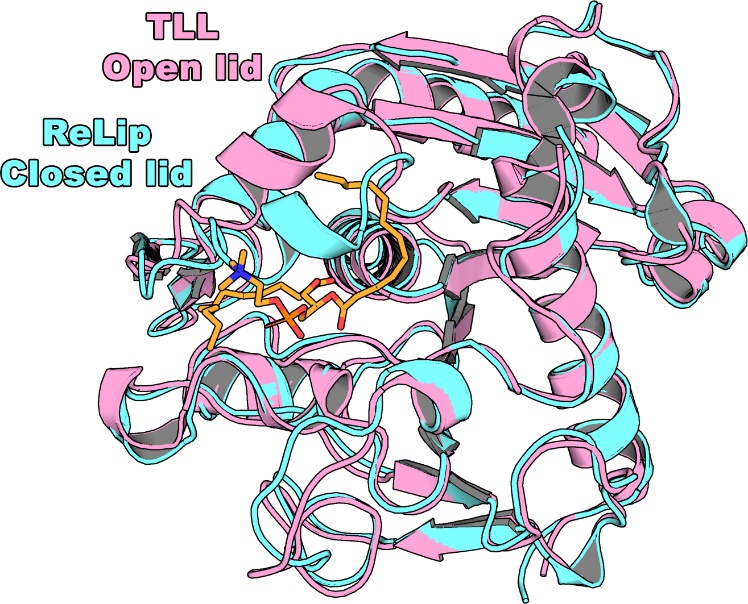


**Figure S7** - Superpositioning of *Re*Lip on TLL in complex with diundecyl phosphatidyl choline (PDB code: 1EIN) highlighting that in the closed conformation of the *lid* in *Re*Lip, the substrate could not be accommodated in the active-site cleft.

**Table S1–** Free fatty acid content and fatty acid profile of vegetable oils (wt%)

| Vegetable oil | Acidity (%) | C8:0 | C10:0 | C12:0 | C14:0 | C16:0 | C18:0 | C18:1 | C18:2 | C18:3 | Others |
| --- | --- | --- | --- | --- | --- | --- | --- | --- | --- | --- | --- |
| Soybean | 2.35 | - | - | - | 0.03 | 13.31 | 2.37 | 22.20 | 58.08 | 3.79 | 0.22 |
| Sunflower | 2.20 | - | - | - | 0.02 | 6.44 | 2.98 | 26.99 | 63.30 | - | 0.27 |
| Corn | 2.24 | - | - | - | - | 15.39 | 1.37 | 33.35 | 49.77 | - | 0.11 |
| Olive | 2.63 | - | - | - | - | 14.79 | 2.24 | 77.45 | 4.66 | 0.15 | 0.15 |
| Coconut | 2.05 | 6.95 | 4.49 | 58.95 | 17.55 | 6.52 | 0.60 | 3.49 | 1.08 | - | - |
| Rapesseed | 2.49 | - | - | - | - | 4.47 | 1.83 | 68.97 | 18.26 | 5.67 | 0.80 |
| Palm | 5.86 | - | - | 0.79 | 0.93 | 51.52 | 3.29 | 37.53 | 5.95 | - | - |
| Macaw | 23.54 | - | - | 0.32 | 0.42 | 21.18 | 1.67 | 67.22 | 8.04 | - | 1.67 |

**Table S2** – Parameters of central composite design, with coded and uncoded levels

| Parameters | Symbol | Coded levels | | | | |
| --- | --- | --- | --- | --- | --- | --- |
|  |  | -1.21 | -1 | 0 | +1 | +1.21 |
| Catalyst concentration (mg/mL)  Time of reaction (hours) | X_C_  X_t_ | 0.5  1 | 0.61  2.2 | 1.15  8 | 1.69  13.8 | 1.8  15 |

**Table S3** – Crystallization conditions

| Method | Robot HoneyBee 963 system |
| --- | --- |
| Plate type | Sitting-drop Corning 3552 |
| Temperature (K) | 291.15 |
| Protein concentration | 16.5 mg.mL^-1^ |
| Buffer composition of protein solution | 50 mmol.L^-1^ phosphate pH 7.4, 150 mmol.L^-1^ NaCl |
| Composition of reservoir solution | 100 mmol.L^-1^ MgCl_2_, 20% PEG 8000, 20% PEG 400, 100 mmol.L^-1^ Tris pH 8.5 |
| Volume and ratio of drop | 1.0 μL, 2:1 |
| Volume of reservoir | 80 μL |

**Table S4** – Data collection, processing and refinement statistics

| **Data collection** |  |
| --- | --- |
| Space Group | *I*422 |
| Cell dimensions |  |
| a, b, c (Å) | 90.98 90.98 115.99 |
| α, β, γ (°) | 90.0 90.0 90.0 |
| Resolution (Å) | 50.00 – 3.00 (3.17 – 3.00) |
| R_meas_ | 21.2 (123.2) |
| *I*/*Iσ* | 15.02 (2.83) |
| Completeness (%) | 99.7 (98.5) |
| Redundancy | 24.41 (26.09) |
| CC_1/2_ | 99.8 (80.0) |
| **Refinement** |  |
| Resolution (Å) | 35.58 – 3.00 |
| N° Reflections | 5129 |
| R_work_/R_free_ | 0.27/0.32 |
| **N° atoms** |  |
| Protein | 1980 |
| Ligand/Ion | 0 |
| Water | 0 |
| **B-factor (Å²)** |  |
| Protein | 79.53 |
| Ligand/Ion | - |
| Water | - |
| r.m.s.d. |  |
| Bond length (Å) | 0.002 |
| Bond angle (°) | 0.537 |
| **Ramachandran Plot** |  |
| Favoured (%) | 93.75 |
| Allowed (%) | 6.25 |
| Outlier (%) | 0 |
| PDB Code | 6UNV |

**Table S5** – Analysis of variance (ANOVA) for response surface quadratic model for the hydrolysis degree of macaw pulp oil, using 95% of confidence interval

|  | Sum of squares | Degrees of freedom | Mean square | F-value | p-value | Significance |
| --- | --- | --- | --- | --- | --- | --- |
| Model | 1360.378 | 5 | 272.076 | 19.29 | 0.000000 |  |
| X_C_ | 217.586 | 1 | 217.586 | 15.43 | 0.007732 | Significant |
| X_C_.X_C_ | 2.981 | 1 | 2.981 | 0.21 | 0.661886 | Not Significant |
| X_t_ | 992.496 | 1 | 992.496 | 70.37 | 0.000156 | Significant |
| X_t_.X_t_ | 147.262 | 1 | 147.262 | 10.44 | 0.017880 | Significant |
| X_C_.X_t_ | 0.053 | 1 | 0.053 | 0.00 | 0.953154 | Not Significant |
| Error | 84.620 | 6 | 14.103 |  |  |  |

X_C_: variable for enzyme concentration and X_t_: variable for time reaction
